# Stroke as an atypical initial presentation of giant cell arteritis

**DOI:** 10.1186/s12877-018-0738-y

**Published:** 2018-02-21

**Authors:** Tharsika Kuganesan, Allen R. Huang

**Affiliations:** 1 Royal College of Surgeons in Ireland school of medicine, 216 Smoothwater Terrace, Markham, Ontario L6B 0M8 Canada; 2 University of Ottawa and The Ottawa Hospital, Civic Campus, 1053 Carling Avenue, Box 678, Ottawa, Ontario K1Y 4E9 Canada

**Keywords:** Giant cell arteritis, Stroke, Atypical presentation

## Abstract

**Background:**

Giant cell arteritis (GCA) is an immune mediated inflammatory disease of medium and large arteries which afflicts older people. The classical presentation features include: headache, visual disturbances, and jaw claudication. Patients diagnosed with GCA have also been observed to be at higher risk for the subsequent development of strokes.

**Case presentation:**

We describe a case of an 84-year old right-handed man who presented to hospital with dysarthria, dysphagia, right-sided facial drop, a history of generalized weakness and multiple falls. He was admitted to geriatric medicine with the working diagnosis of a posterior circulation stroke syndrome. He was also started on antibiotic treatment for a possible community-acquired pneumonia because of the presence of a low-grade fever and a chest radiograph showing ill-defined left lower lobe airspace disease. Initial lab results were remarkable for an erythrocyte sedimentation rate (ESR) of 112 mm/h and a C-reactive protein (CRP) level of 110 mg/L consistent with an active inflammatory state. Neurovascular imaging showed mild atherosclerotic changes of the aortic arch and proximal great vessels without significant stenosis. The patient was started on daily high-dose prednisone because of the possibility of a cerebral vasculitis. Bilateral biopsy of temporal arteries showed giant cell arteritis. The patient’s neurologic status and inflammatory markers significantly improved (ESR 52 mm/h, CRP 7.0 mg/L) and he was eventually discharged to a seniors home with services.

**Conclusion:**

The initial presentation of giant cell arteritis as a stroke syndrome, especially in the posterior circulation territory, is exceedingly rare. Other atypical presenting symptoms may include chronic cough and fever of unknown origin. The elevated ESR and CRP levels were clues to the diagnosis and clinical decision-making should be driven by a high index of suspicion since no single test (ESR, CRP, temporal artery biopsy) has perfect sensitivity. Elevated CRP may have a role in increasing stroke risk. This case report illustrates that in older people clinicians must consider atypical presentations of disease more often since timely diagnosis and initiation of treatment can result in optimal outcomes.

## Background

Giant cell arteritis (GCA) is an immune mediated inflammatory disease of medium and large arteries [1, 2]. Typically, GCA presents in patients over 50 years old with headache, scalp tenderness, jaw claudication, and sudden vision loss. GCA is commonly associated with polymyalgia rheumatica, which presents with symptoms of aches and pains in the shoulders, neck and hips. Patients with GCA are at higher risk for developing subsequent strokes [3–5]. There are some instances where GCA initially presents atypically as described in the case below.

## Case presentation

An 84-year old right-handed male presented to hospital with dysarthria, dysphagia, right-sided facial drop, a history of generalized weakness and multiple falls.

The patient had a past history of a transient ischemic attack (TIA) 8-years prior to presentation, manifesting as speech disturbance and weakness, type 2 diabetes mellitus, hypertension, and glaucoma. His medications on presentation included: perindopril 8 mg daily, amlodipine 5 mg daily, clopidogrel 75 mg daily, metformin 500 mg b.i.d., amitriptyline 25 mg qHS and timolol ophthalmic drops 0.5% - 1 drop each eye b.i.d.

He was a retired electrician and had quit smoking 35 years ago. He did not drink alcohol. He lived alone, independently in his own home. He usually walked using a cane. He received meals delivery services and his niece assisted him by driving him to where he needed to go.

The patient had an unwitnessed fall in his home and subsequently activated his emergency response device. He was brought to the Emergency Department by the emergency medical services. He was alert on arrival and was noted to have speech difficulties. His vital signs showed a low-grade fever of 38.5 °C oral and an elevated heart rate of 105 beats per minute. His clinical exam showed that he was alert, with new dysarthric speech, a pronator drift of his right arm and a fine tremor of his left hand. Ausculation of his chest was clear. Initial laboratory results are shown in Table 1. Remarkable results include an elevated erythrocyte sedimentation rate (ESR) of 112 mm/h (normal 0–6) and a C-reactive protein (CRP) of 110 mg/L (normal less than 10). A chest radiograph was reported as showing ill-defined left lower lobe airspace disease, possibly representing a pneumonia. A plain computed tomography (CT) of his head showed moderate cerebral parenchymal atrophy and chronic periventricular microvascular ischemic changes. The patient was hospitalized and admitted to the Geriatric Medicine service. He was given intravenous azithromycin followed by oral cefuroxime for a presumed community-acquired pneumonia. The neurology service suggested additional brain imaging with magnetic resonance scan (MRI) and a CT angiogram of his head and neck to further clarify the etiology of this man’s possible stroke syndrome. At no point during this person’s multiple clinical evaluations did he report headache, visual problems, or jaw claudication. The CT angiogram of the neck and circle of Willis showed mild atherosclerotic changes of the aortic arch and proximal great vessels without significant stenosis. There was a pseudoaneurysm of the mid-cervical portion of the right internal carotid artery and mild luminal irregularity of the vertebral arteries bilaterally at the level between the 1st and 2nd cervical vertebrae. The radiologist was unable to differentiate between vasculitic or atherosclerotic changes in the vertebral arteries and suggested further imaging. Magnetic resonance arteriography was unable to be performed due to the presence of a metallic foreign body embedded in his left eye. An echocardiogram showed normal cardiac function and normal valve anatomy and function. A 48 h Holter recording showed sinus rhythm, with very rare premature atrial and ventricular beats. Atrial fibrillation was not detected. His repeat inflammatory markers 5-days after admission were: ESR 90 mm/h, CRP 47.7 mg/L. In view of a possible cerebral vasculitis the patient was started on prednisone 60 mg daily. Bilateral temporal artery biopsies were done on the 7th hospital day. A contrast-enhanced CT of the thorax, abdomen and pelvis was done to investigate the possibility of diffuse vasculitis and these studies were reported to show no evidence of aortitis or medium and large artery vasculitis.

**Table 1:** Initial lab results

Laboratory Results (SI units; conversion units; normal range)	
Sodium	137 mmol/L; (137 meq/L); 136-145 mmol/L
Creatinine	89 μmol/L; (1.01 mg/dL); 49-93 μmol/L
Urea	9.2 mmol/L; (25.76 mg/dL); 2.1–8.0 mmol/L
Serum glucose	7.8 mmol/L; (141 mg/dL); 4.0–11.0 mmol/L
Hemoglobin	113 g/L; (11.3 g/dL); 125–170 g/L
WBC	10.9 × 10^9^/L; 3.5–10.5 × 10^9^/L
Creatine kinase	615 U/L; 30–250 U/L
C-reactive protein (CRP)	110 mg/L; less than 10.0 mg/L
Erythrocyte sedimentation rate(ESR)	112 mm/h; 0–6 mm/h
Serum iron	5 μmol/L; 12–31 μmol/L
Iron saturation	19%; 20–50%
Ferritin	479μg/L; 24-336μg/L

**Fig. 1:**
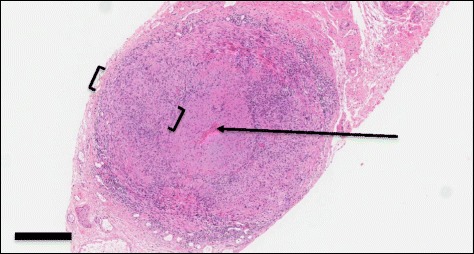
Photomicrograph of a hematoxylin and eosin stained section of the temporal artery biopsy, showing transmural inflammation (square brackets). The arrow indicates the arterial lumen. The solid bar represents **400** μm

**Fig. 2:**
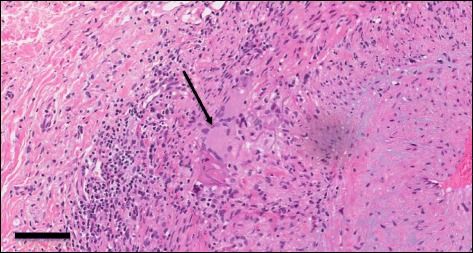
Close-up of a multi-nucleated giant cell (arrow). The solid bar represents **100** μm

The biopsy results (right artery specimen length 1.9 cm, left 2.0 cm) were reported on the 10th hospital day and was positive for GCA showing transmural inflammation and the presence of multi-nucleated giant cells in the arterial wall (Figs. 1 and 2). The prednisone dose was maintained at 60 mg daily. After 2-weeks methotrexate 10 mg weekly was added, with the plan to slowly taper down the prednisone dose and increase the weekly methotrexate dose. The patient’s dysarthria, dysphagia and right arm weakness progressively improved. He was able to swallow normally and his speech became clear. He regained safe independent mobility using a 4-wheeled walker and was discharged to a seniors home with services on the 29th hospital day.

Table 2 shows the serial results of his serum inflammatory markers.

**Table 2:** Serial inflammatory marker results

Time	ESR (mm/h)	CRP (mg/L)
Admission	112	110
Day 5 (prednisone 60 mg daily started)	90	47.7
Day 6	75	34.6
Day 7	80	21.6
Day 10	52	7.0

## Discussion and conclusion

A comprehensive but not systematic review of the literature found that people with the diagnosis of giant cell arteritis were at higher risk for the subsequent development of stroke [3, 6–9]. A review by Napoli postulated a causal link between the increased production of CRP in an inflammatory condition such as GCA and the development of stroke [10]. To our knowledge this is the first case where the initial presentation of GCA is a posterior cerebral circulation stroke syndrome. Other case reports of atypical presentations of GCA are rare. One case described a patient over 50 years of age who presented with a 2-month history of chronic cough and fever of unknown origin with an elevated ESR, which was confirmed to be GCA by temporal artery biopsy [11]. Approximately 10% of GCA patients have upper respiratory tract infection symptoms [12]. Other respiratory symptoms that could present in GCA are sore throat and hoarseness [11]. The patient described in our case did present with a fever but had no respiratory symptoms. He was treated for a possible pneumonia because of the chest radiograph findings.

An atypical presentation of GCA could delay diagnosis and treatment and therefore increase the risk for complications specifically irreversible vision loss. There can be a variety of symptoms that can be present in GCA, however in most cases the ESR will be persistently elevated. High clinical suspicion should always drive decision-making since diagnostic testing have their limitations. The sensitivity of an elevated ESR is 84% and CRP is 87% and both have a specificity of 30% [1, 13]. Temporal artery biopsy has a reported sensitivity of 70 to greater than 90% [2, 14] which still leaves a small number of patients with GCA who have biopsy-negative results. One study reported that combining ESR and CRP results generated a specificity of 97% for GCA [15]. Imaging to detect arterial inflammation by positron emission tomography as suggested by a recent systematic review and meta-analysis may be helpful, although the accessibility of this specialized technique would limit its general use [16].

In conclusion although there is a set of typical symptoms associated with giant cell arteritis, such as headache, scalp tenderness, jaw claudication, and visual disturbances, atypical symptoms in patients older than 50-years should also be considered for GCA. These atypical symptoms may include chronic cough, fever of unknown origin, and stroke symptoms especially if the patient’s ESR levels are elevated. Clinical decision-making should be driven by a high index of suspicion since no single test has perfect sensitivity. Whether a combination of ESR, CRP and temporal artery biopsy results can improve the diagnostic accuracy of GCA would need further study. As with all conditions, prompt diagnosis and the timely initiation of targeted treatment improves the chances of optimal outcomes.

## Abbreviations

CRP: C-reactive protein
CT: Computed tomography
ESR: Erythrocyte sedimentation rate
GCA: Giant cell arteritis
MRI: Magnetic resonance imaging
TIA: Transient ischemic attack
